# Test-retest reliability and concurrent validity of knee extensor strength measured by a novel device incorporated into a weight stack machine vs. handheld and isokinetic dynamometry

**DOI:** 10.1371/journal.pone.0301872

**Published:** 2024-05-22

**Authors:** Pradeep K. Sahu, Noel Goodstadt, Arun Ramakrishnan, Sheri P. Silfies

**Affiliations:** 1 Department of Exercise Science, Arnold School of Public Health, University of South Carolina, Columbia, South Carolina, United States of America; 2 Department of Physical Therapy and Rehabilitation Sciences, Drexel University, Philadelphia, Pennsylvania, United States of America; 3 College of Nursing and Health Professions, Drexel University, Philadelphia, Pennsylvania, United States of America; Faculty of Health Sciences, University of Primorska, SLOVENIA

## Abstract

**Background:**

The current clinical gold standard for assessing isometric quadriceps muscle strength is an isokinetic dynamometer (IKD). However, in clinics without an IKD, clinicians default to using handheld dynamometers (HHD), which are less reliable and accurate than the IKD, particularly for large muscle groups. A novel device (ND) was developed that locks the weight stack of weight machines, and measures forces applied to the machine, turning this equipment into an isometric dynamometer. The objectives of this study were to characterize the test-retest reliability of the ND, determine the within-day and between-days inter-rater reliability and concurrent validity compared with that of the HHD, in healthy volunteers (HV) and individuals with knee osteoarthritis (OA) for measuring knee extensors isometric muscle force.

**Materials and methods:**

29 healthy (age = 28.4 ± 7.4 years) and 15 knee OA (age = 37.6 ± 13.4 years) participants completed three maximum force isometric strength testing trials on dominant side knee extensor muscles on three devices (ND, HHD, and IKD) in two separate sessions by two raters. The maximum force (Fmax) produced, and the force-time series were recorded. Reliability and validity were assessed using Intraclass Correlation Coefficient (ICC), Bland-Altman Plots, Pearson’s r, and cross-correlations.

**Results:**

The ND demonstrated excellent test-retest reliability (ICC_2,3_ = 0.97). The within-day (ICC_2,3_ = 0.88) and between-day inter-rater reliability (ICC_2,3_ = 0.87) was good for HHD. The ND showed excellent within-day (ICC_2,3_ = 0.93) and good between-day (ICC_2,3_ = 0.89) inter-rater reliability. The Bland-Altman analysis revealed HHD systematic bias and underestimation of force particularly with quadriceps force values exceeding 450 N. Mean differences were found in maximum force between HHD vs. IKD (MD_abs_ = 58 N, p < .001) but not the HHD vs. ND (MD_abs_ = 24 N, p = .267) or ND vs. IKD (MD_abs_ = 34 N, p = .051). The concurrent validity of Fmax (r = 0.81) and force-time curve correlation (0.96 ± 0.05) were the highest between the ND and IKD.

**Conclusions:**

The ND’s test-retest reliability and concurrent validity make it a potential strength assessment tool with utility in physical therapy and fitness settings for large muscle groups such as the knee extensors.

## Introduction

Knee joint injuries account for 39.8% of all sport and 60% of high-school sport-related surgeries in the United States (US) [1, 2]. Quadriceps strength is critical in preventing knee injuries and degenerative joint disease by attenuating ground reaction force [3, 4]. Quadriceps strength impairment is common in osteoarthritis (OA) and post-knee surgery, with deficits that can persist even for years, impacting the ability to perform lower extremity function and sports activities [4–6]. Additionally, longitudinal data indicates quadriceps strength is a robust predictor of functional decline and mortality due to coronary artery disease and chronic obstructive pulmonary disease [7, 8]. Studies showed a positive association of quadriceps strength with dynamic balance, gait speed, stride length, fall risk, general health, and function [9–13]. Evaluation of peak muscle force (strength) is an essential clinical component of assessing muscle function [14]. Moreover, quadriceps strength assessment helps quantify strength deficits, intervention program effectiveness, progression through rehabilitation evaluation for return to sport, and development of normative data [12, 13, 15–17].

Objective strength measurement tools used in clinical practice vary from hand-held dynamometers (HHD) to computerized isokinetic dynamometry (IKD). Utilizing any device on a large scale for clinical practice depends on several factors, such as reliability, validity, affordability, safety, and portability [18]. Reliability evaluates the consistency of repeated measurements, and test-retest reliability specifically addresses consistency across measurement sessions using either the same (*intrarater*) or different (*interrater*) clinical testers [19]. Validity is defined as how well the device measures what it intends to measure. Concurrent validity assesses how well a new device measurement matches an existing criterion.

In clinical practice, muscle strength is often assessed with a HHD [20]. When evaluating knee extensor strength, the examiner typically stabilizes the HHD against the distal anterior tibia, (manually or assisted by a belt) to resist the participant’s active knee extension and record the highest amount of force produced during the test [21]. HHDs are portable, inexpensiveness (∼$1000), and user-friendly, which supports their use in physical therapy, sports, and fitness industries. When larger muscle groups are assessed, HHD application requires changes in practice to account for greater stabilization requirements (for muscles producing more than 15–30 kg force), and alteration in examiner or client position to reduce inconsistency and inaccuracy [21, 22]. Variability in measurement practice is especially problematic for large muscle groups such as knee and hip extensors, as forces produced can reach or exceed 500 newtons [23]. These larger forces rely on the tester’s ability to manually resist a patient-generated force; thus, during the use of a HHD, the tester’s ability can affect both the reliability and validity of an isometric force reading [21, 24, 25]. Studies have reported differences in maximum force measures of knee muscle extensors and flexors between male and female testers, with the study by Kelln et al. reporting lower reliability with female testers compared to males [26, 27]. To eliminate the issues related to HHD use, the current gold standard for assessing large muscle group isometric strength is the IKD. IKDs are large pieces of equipment that control body segment stabilization, positional consistency, and resistance without the need for the assessor to provide them. Research documenting the reliability and validity of these devices makes them the gold standard for strength testing [28, 29]. Additionally, IKD can measure various variables, including torque, power, and velocity, and the capacity to maintain patient data over an extended period [30]. While a number of companies produce these devices (e.g., Biodex, Computer Sports Medicine Inc., BTE Technologies), they are expensive (>$40,000) and the associated space requirements restrict the availability of IKDs and often limit their use to research and elite sports training facilities [24, 31]. The IKD can be cumbersome to set up for testing, and training is needed for the efficient operation of the device, further challenging its application.

Reported *intrarater* reliability is moderate to good using conventional HHD protocols for assessing knee extensor muscle strength (examiner provided resistance against a client-generated force). However, *interrater* and test-retest reliability, an indicator of a device’s practical use in a clinical setting, is unclear, and significant variations are detected in strength measurement between testers and sessions [27, 32, 33]. Prior work has modified HHD protocols to reduce the influence of the tester’s ability to generate an adequate counterforce. Approaches include the use of belts looped around the back leg of the chair to anchor the HHD [34]. Other studies focused on controlling the influence of the tester within the protocol to reduce the error created by poor stabilization of the person or position (providing support by belt and chair frame) [35–38] and leveraging resistance (dynamometer mounted either on a PVC pipe or the back of the chair with a belt) [39, 40]. However, these controlled studies rarely address *interrater* or test-retest reliability. Without between-day test-retest reliability, it is impossible to objectively determine if a client is truly improving their strength in response to an intervention.

Although the modified HHD and force transducer systems have several advantages related to the costs, the major drawbacks in those studies were poor fixation of the HHD pad on the tibial segment [35–44] and lack of proximal stabilization [36–40, 42, 45–47]. Further, many HHDs do not provide feedback during testing [35, 37–41, 43–46], are time-consuming for setting up participants [35, 36, 42], and are not completely tester hands-free measurements [35, 38–41, 43]. A few modified HHD systems are not portable [35, 36, 41, 42, 46, 47], and measuring the strength of other large muscle groups is not provided by the equipment [35, 41, 45–50]. During strength testing, the contact surface of the pad on the tibia may produce pain or discomfort, as reported in a paper [34]. Therefore, it may produce lower force, especially in ongoing painful individuals.

HHD validity studies compared to IKD report correlation values from .43 to .99 across arm and leg muscles, with inconsistent evidence for validation for larger muscle groups [51, 52]. Experiments conducted using modified HHD protocols (external stabilization of the device or person) also provide conflicting results, with some studies indicating that HHD provides a lower force value than IKD, while another study reports overestimation [37, 43]. Despite varying protocol modifications, no standardized protocol has been established that allows clinicians to implement an optimal test efficiently for larger muscle groups. The discussion of limitations of the use of HHD noted in prior studies, particularly when measuring large muscle group strength, suggests the need for adaptations to testing that allow for more consistent setup, stabilization, and measurement or the necessity for the expensive IKD.

To this end, a novel isometric strength testing device (ND; Patent US 11,099,089) was constructed to address the limitations of the HHD and the current gold standard (e.g., cost, portability, size) while providing reliable and valid strength measures of larger muscle groups. The ND locks down any weight stack, converting it into an isometric system and allowing measurement of the forces applied by the individual through the weight stack cable. Weight stack machines provide improved customization, standardization, and stabilization for individuals during strength testing and eliminate tester manual resistance, and stabilization errors (see the Materials and methods section for details).

Therefore, the first objective of this study was to determine the within-day test-retest reliability of the ND to assess knee extensors’ isometric muscle strength in healthy volunteers (HV) and individuals with knee osteoarthritis (OA). The second objective was to find the within-day and between-day *interrater* reliability of the ND, and to compare it with that of the HHD (with rater manual resistance and participant stabilization). The third objective was to determine the concurrent validity of the ND against the gold standard (IKD) and differentiate that from the concurrent validity of the HHD.

## Materials and methods

### Participants

This was a prospective observational study conducted at Drexel University. The detailed protocol was approved by the University Institutional Review Board for human subjects (IRB 1610004896), and the participants provided their written consent after discussing potential risks. The sample size was calculated based on the formula reported by Walter et al. for reliability studies (Online calculator-https://wnarifin.github.io/ssc/ssicc.html) [53, 54]. To demonstrate the test-retest reliability of ND with expected reliability (ICC = 0.85) and a confidence interval (CI = ± .1), 80% statistical power, and two measurements, we calculated the minimum sample size required as 31. We estimated the test-retest reliability of ND to be at least 0.85 as published studies using an HHD demonstrated the *intrarater*, test-retest ICC of knee extensors is 0.78. We believed the ND would surpass this ICC value [21]. We adjusted the sample size needed for this study to 33 participants to account for a 10% dropout rate.

Flyers and posters in the local community and at the university were used to recruit healthy participants (no knee injury or pain) and those diagnosed with knee OA. Participants were recruited between January 3 and October 6, 2017. An online survey, email, and telephone interviews were conducted by the investigators to confirm the participant’s eligibility. The inclusion criteria for participants were adults between 18 to 60 years old. The healthy participants had to report no lower extremity injury and no diagnosis of knee or hip OA. Participants with knee OA had a diagnosis of mild to moderate knee osteoarthritis in their dominant leg, were able to walk and climb stairs without an assistive device and had no activity restriction by a physician (cleared for exercise). Potential participants in both groups were excluded if any current or previous spine or lower extremity injury, that significantly affected their daily activities, or recent (last three months) knee or hip surgery on the dominant lower extremity. Other exclusion criteria were neurological deficits, altered sensation, diagnosed depression or anxiety, fatigue disorders, or chronic widespread pain that could affect force production and fatigue of the muscle.

### Novel device

**Fig 1:**
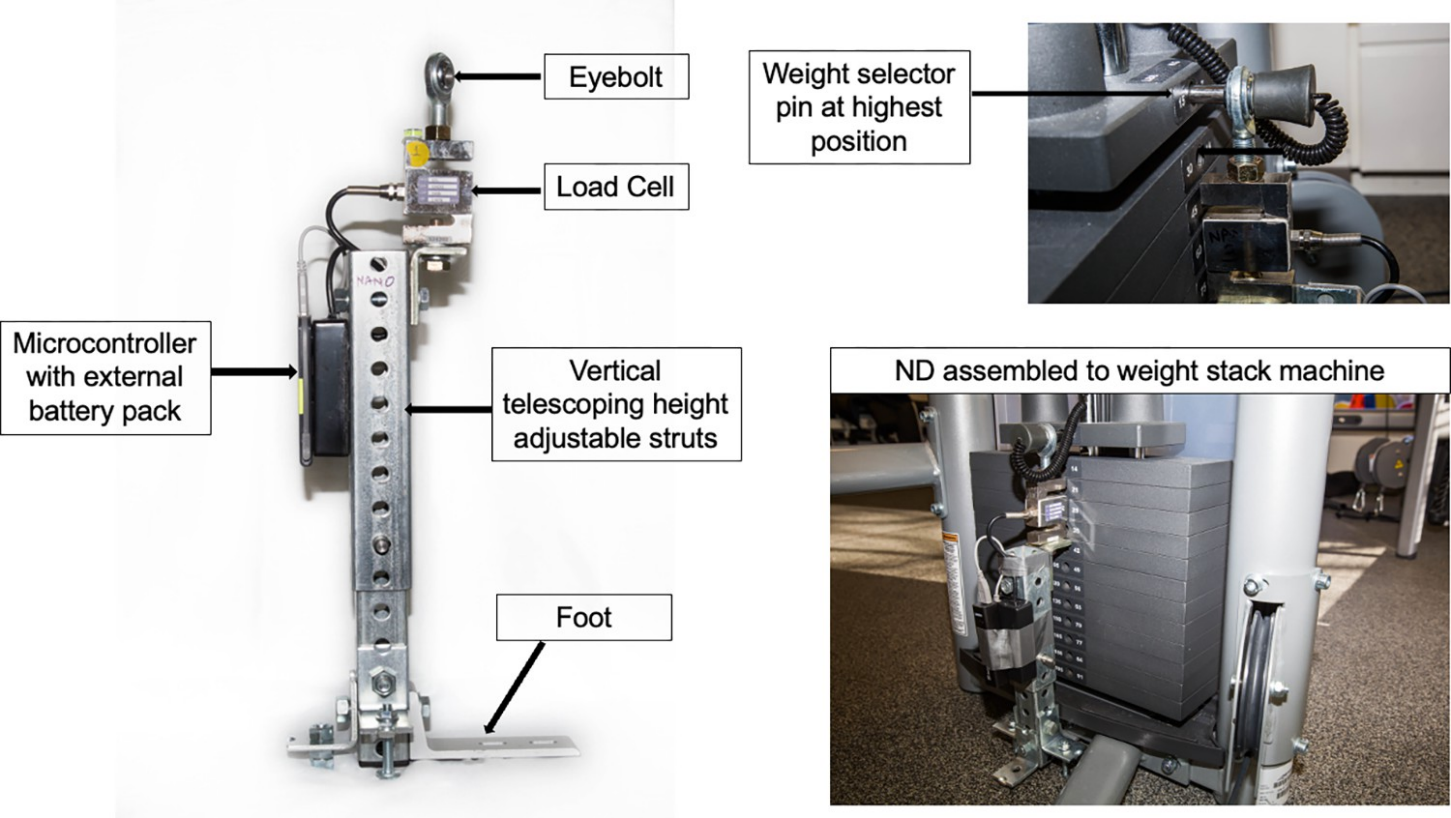
Components of the novel device. The left figure shows the configuration of different parts of the novel device, as described above. The device can be anchored under the bottom of the steel frame of any commercially available weight machine (right bottom figure). The top of the strut has an eyebolt through which the weight selector pin passes and is set at the highest weight stack (right top figure). This locks down the weight stack, converting it into an isometric system and allowing measurement of the forces applied by the individual to lift the weight stack. The load cell registers the application of a tension force to the steel pin. The force is sensed by the digital-to-analog microcontroller attached opposite the load cell. Real-time force data, including the force-time graph from the microcontroller, can be wirelessly displayed and recorded by associated custom software.

The ND consists of structural and electronic elements (Fig 1). Structural elements are composed of sliding telescopic struts that form the vertical post on which we fixed a 6” right angle foot on the bottom that slides under the weight stack machine’s frame. A 1” right angle bracket is secured on the top telescoping strut in which we mount the S-beam load cell (AmCells STL-500lb, tacunasystems.com). An eyebolt was fitted on the top of the load cell, which served as the guide for the pin that locked the weight stack. The electronic components consist of a load cell amplifier (HX711 with 5V excitation) connected to a microcontroller (ATmega328P Nano Board, ARDUINO). Custom firmware was programmed into the microcontroller, allowing it to read the sensor output, convert it to newtons, and transmit it via Bluetooth (HC06) RS232 serial port protocol to a laptop running our custom software. To power these electronics, our initial design used an external 5V rechargeable battery pack, which was later swapped with a 7.4V internal Li-Poly battery and charger pack. A custom graphical user interface (GUI) program running on the laptop/tablet was written in LabVIEW (version 8.6, National Instruments, Austin, TX) to connect, display, and save real-time data. A PC application was developed that added functionality, including subject profile, report generation, and several graphing improvements. For additional information regarding the development and design of the device, see https://doi.org/10.17605/OSF.IO/E4GWF.

### Procedures

**Fig 2:**
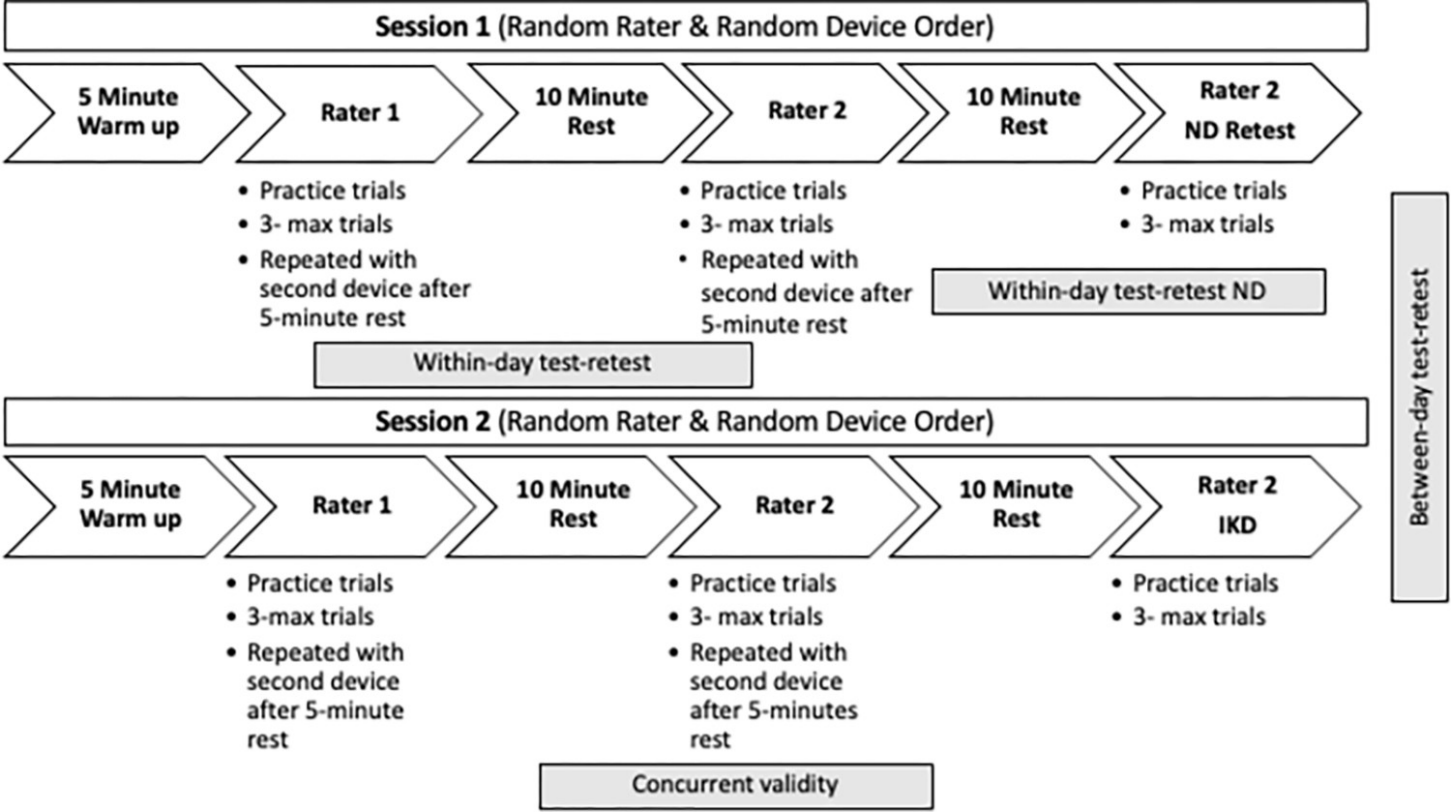
Measurement protocol. This is a schematic of the two-session testing protocol, with an indication of the datasets used for each analysis.

Fig 2 provides an overview of the measurement protocol, which was conducted in two separate sessions 3–14 days apart. The total time needed to complete each session was approximately 1.5 hours. Session one included: assessment of demographics and anthropometric data [age, sex, height (cm), weight (kg), thigh and lower leg segment lengths (cm), and leg dominance (the leg used to kick a soccer ball with maximal effort)]. Participants completed the Saltin-Grimby Physical Activity Level Scale (SGPALS) questionnaire [55]. This is a 4-level scale that measures leisure time physical activity, with the lowest score ‘of 1’ representing “physically inactive” and the highest score ‘of 4’ representing “regular hard physical training for competitive sports.” This tool previously showed a positive association with cardiovascular risk factors, mental health, and predictors of cerebrovascular diseases [56–58]. This self-reported scale has demonstrated good reliability and validity in a large-scale adult population [59]. The participants with knee OA were also asked to note their side of pain and the intensity of pain recorded using an 11-point numeric pain rating scale (NPRS) [‘0’ corresponding to “no pain” to ‘10’ represents “maximum intensity of pain”]. This scale has demonstrated acceptable ranges of reliability [60]. During each session, raters collected dominant leg knee extensor muscle strength using the HHD and ND with device testing and rater order randomized *a priori*.

In session one, the second rater completed a second measurement series using the ND to obtain the ND’s test-retest reliability. The strength measurement using the IKD was always performed in the second session and by the second rater after the measurements using the ND and HHD were completed. Two licensed physical therapists and one exercise scientist (three males) were trained as testers on all three devices. All testers were familiar with and used HHD and IKD testing devices.

### Pre strength-testing set-up

The participants were asked to wear shorts. They were positioned in the knee extension weight stack machine, and the axis of the device was aligned with the participant’s knee axis (dominant leg). The shin pad was kept as low on the tibia as possible, proximal to the lateral malleolus, with the participant still able to dorsiflex their ankle to a neutral position. Anthropometric measurements were taken in this position: thigh length (greater trochanter to lateral femoral condyle), leg length (knee joint line to the marked point for tibial pad), tibial pad location (mid-point on lateral malleolus to the tibia pad mark) and used to standardize the location of resistance on the distal tibia across the devices.

The participants performed 5 minutes of cycling on a stationary bike (75–100 watts), with no resistance, for warm-up before conducting measurements. As part of the warm-up, the participants performed one sub-maximal trial (50–75% of possible maximum effort) and 1–2 trial(s) with maximum intensity on each device. During the warm-up and practice trials, participants were instructed to build their force gradually and then hold the maximum effort. The instruction was “once the tester says start, build the force to reach the maximum effort in 1–2 seconds and hold the maximum until the tester says stop” (which was standardized to 5 seconds)”. No visual or verbal feedback was provided during warm-up or testing, and a 5-second contraction time was used to obtain a maximum force value [61]. A one-minute rest was provided between the sub-maximal and maximum trials. Then, three maximum isometric force (Fmax) trials were performed with a 1-minute rest between the trials to avoid fatigue [62]. A 5-minute rest period was given between the devices and 10 minutes between the raters (Fig 2).

### Testing protocol using the HHD

**Fig 3:**
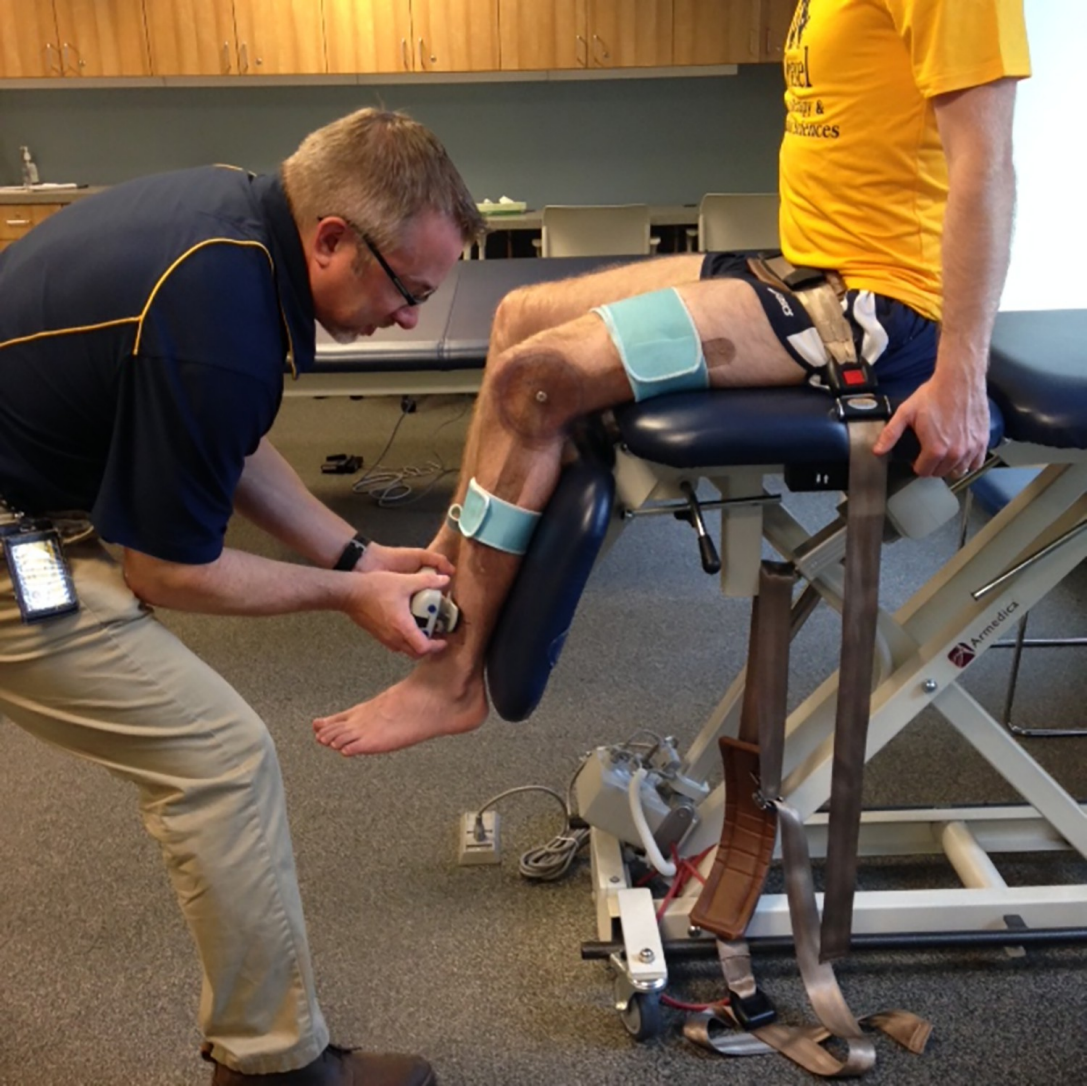
Set up for HHD testing. Photo of the set-up for measuring isometric knee extensor strength on the dominant side using a commercially available HHD (Lafayette Instruments, US, model no. - 01165). The participant was positioned in high sitting at the end of a treatment table with legs hanging off the side at 90⁰ of knee flexion and posterior thigh in complete contact with the treatment table. The proximal thigh was fixed to the treatment table by a mobilization belt. To ensure the angle of the knee joint was at 90⁰ before each trial, a universal goniometer was attached using Velcro with the axis at the lateral femoral condyle, fixed arm directed towards the greater trochanter, and movable arm towards the lateral malleolus. The HHD units were set to Newtons (N) and measurement time to 5 seconds before placing the device on the participant’s tibia. The HHD is strapped to the tester’s dominant hand so that the tester can place the measurement side of the HHD on the previously marked point of the participant’s tibia. The plinth height was raised to allow the tester to stabilize their elbow at 90 degrees of flexion against their body when providing resistance perpendicular to the tibia against the participant’s knee extensor force.

After the participants and the HHD were set up for testing (Fig 3), the participants were instructed to keep their trunks straight and hold the sides of the plinth to avoid any backward movement of the trunk. HHD force application by the testers was at a right angle to the tibia. Before starting the measurement, the HHD force was reset to zero. A second investigator recorded the maximum force from the HHD screen, and the time-series data were recorded at 40Hz.

### Testing protocol using the ND

#### Set-up of the ND

Attaching and anchoring the ND to the weight stack requires aligning the device with the center of the weight stack and sliding the adjustable base under the frame of the weight machine. The device should be lifted so the adjustable base of the unit is in contact with the base of the weight machine frame prior to pushing the weight stack pin through the eyebolt of the ND. Gross adjustment to align the eyelet with the hole of the top plate on the weight stack is through a pin and slide mechanism. Fine adjustment is via a nut attached to the eyelet to adjust the height within fractions of an inch for the pretension of the load cell. The entire process takes less than 5 minutes. Further procedures to run the test require the patient being fitted into the machine to align the joint being tested to the axis of the machine lever arm, measuring the distance between the axis of rotation and the point of force development, and setting up the test information required. Test information includes auto-generated patient ID, test type and the number of trials, notation of the involved side, patient’s diagnosis, test limb, and joint angle. Other information that can be saved is turning on enabling of threshold and percentage of threshold and providing notes on set-up or other important information. Once saved, the Bluetooth is connected, and the test is ready to run.

#### Testing by using ND

**Fig 4:**
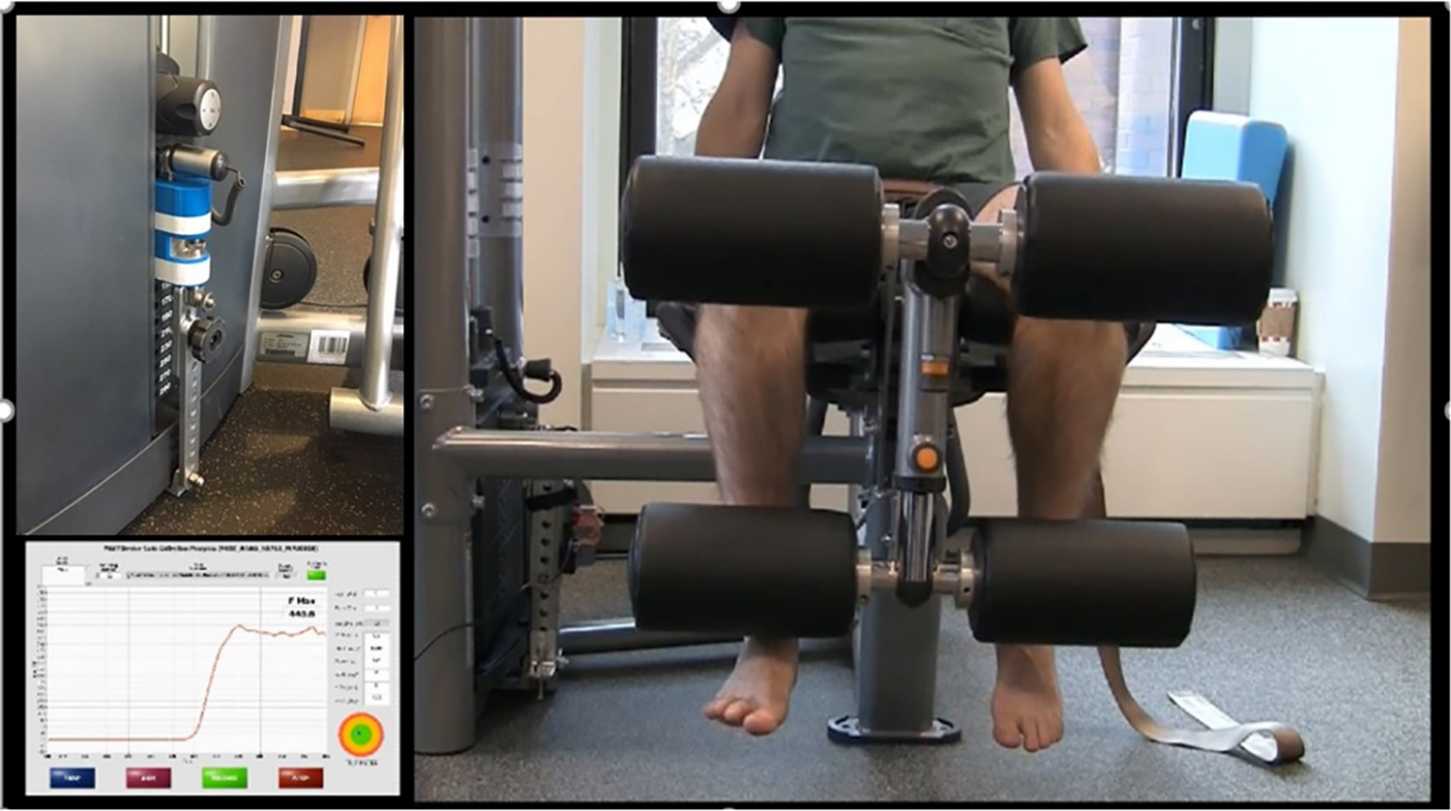
Set up for ND testing. Photo of the set-up testing knee extensor strength with the ND (left top panel) and the accompanying generated force-time curve (left bottom panel; X-axis is time, and Y-axis is the force in newtons). Participants were seated in the knee extension weight stack machine (Life Fitness, Illinois, USA; right panel). The axis of the participant’s knee joint (tip of lateral femoral condyle) and the machine axis were aligned. Participants were positioned so that the seat fully supported their thighs, and pillows were used when necessary to ensure a trunk-neutral position. The strapping, belt fixation, and goniometer placement procedures were the same as the HHD protocol. Knee flexion was aligned to 90⁰, and participants were asked to hold the side handles of the chair similar to the HHD protocol to maintain the neutral trunk alignment. The tibial pad was located at the mark on the tibia. The ND was inserted into the weight stack, leveled, and adjusted so no slack was present in the cable system.

Once the participants and the ND were set (Fig 4), the load cell force output was set to zero. The force data were collected by the custom-built software at 10 Hz. The software was set to account for the machine’s CAM design at 90 degrees of knee flexion. The second investigator recorded the maximal force values, and time-series data were recorded.

### Testing protocol using the IKD

**Fig 5:**
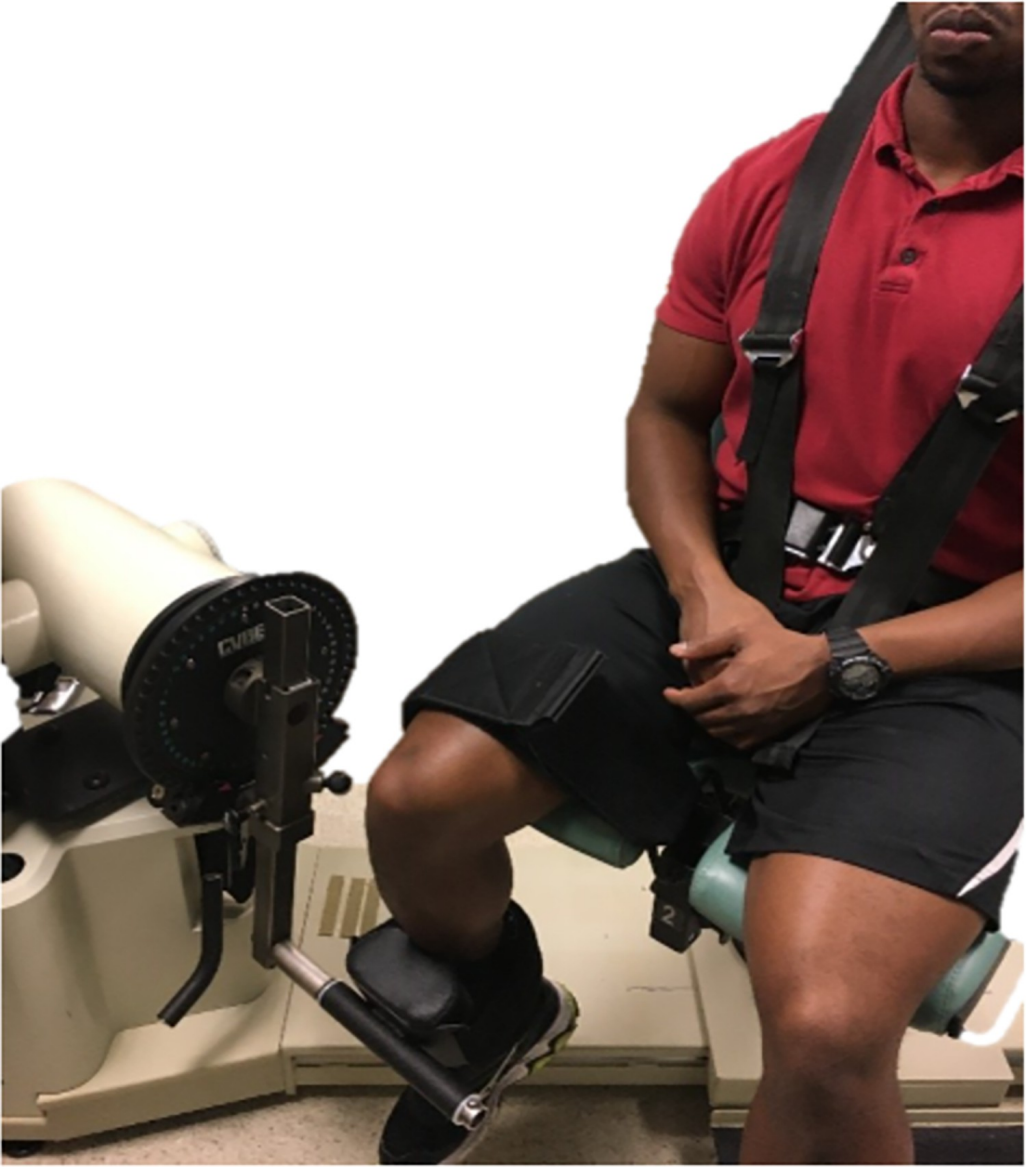
Set up for IKD testing. Photo of the set-up for testing knee extensor strength using the IKD. The participants were seated in the IKD machine chair (Cybex, Humac Norm, US), with the lateral epicondyle of the tested side femur centered with the IKD’s input arm axis by adjusting the chair’s position. The hip knee adapter stabilizing pad was kept against the participants’ tibia at the tibial marking by adjusting the knee/hip adapter and stabilizing pad until the bar was at the correct height. The investigators strapped the torso, thigh, and tibial harness to provide stability during the test. The knee angle (90⁰ of flexion) was manually adjusted using the software and confirmed with a universal goniometer.

The participants were asked to sit in the IKD machine chair (Cybex, Humac Norm, US; Fig 5). Force was recorded (kg), and the device was set to isometric mode with maximum force recorded and time-series data collected at 100 Hz. Fmax and time-series data were converted to Newtons (N) for analysis.

### Statistical analyses

Statistical analysis was conducted using the SPSS version 28 (Armonk, NY: IBM Corporation). Demographics (Mean, SD, N) were calculated for participants’ general characteristics. The normality of data was checked by the Shapiro-Wilk test. The average value (Mean, SD) of three maximum force trials for each test was used for analyses. Data were analyzed as all participants combined and separately by group (HV, OA). Session one data was utilized for within-day *interrater* reliability analyses and ND reliability (*intrarater*, second rater data) using a two-way mixed random absolute agreement model (ICC_2,3_). Data from both sessions was used for *interrater* between-day reliability of the HHD and ND using a two-way random model absolute agreement (ICC _2,3_) [63]. ICC values were interpreted as follows: < 0.5; poor reliability, 0.5 to 0.75; moderate reliability, 0.75 to 0.90; good reliability, and > 0.90; excellent reliability [64]. The standard error of the mean assessed absolute reliability (SEM) = SD_pooled_ × √ (1 –ICC), where SD_pooled_ is the mean of both SDs. Minimal detectable difference (MDD) was calculated using the formula MDD = SEM × z score × √2, and the MDD 95% (z score = 1.96).

The data from the second testing session were used to analyze concurrent validity. To assess systematic bias, Bland-Altman plots, including the limits of agreement (LoA), were created between each pair of devices for all participants, and HV and OA groups separately in MATLAB (The MathWorks, Inc., Natick, MA, USA) [65]. One-way repeated measure ANOVA was implemented to compare the maximal force value across the three devices, and a pairwise comparison was performed to obtain the differences between each pair of devices. Pearson correlations (r) of maximum force values between each pair of devices were calculated and interpreted as negligible (0.0–0.10), weak (0.10–0.39), moderate (0.40–0.69), strong (0.70–0.89) or very strong correlation (0.90–1.00) [66]. Force-time curves representing force generation patterns between each pair of devices for each participant were down-sampled to 10 Hz, spline interpolated, and correlated in MATLAB. All statistical analyses were two-tailed, and the significance level (p) was < 0.05.

## Results

### Demographics and general characteristics

Twenty-nine HV (age: 28±7) and 15 OA (age: 38±13) participants (total n = 44) were deemed eligible and participated in this study. For the reliability analysis, six healthy individuals’ data were excluded from the study due to device errors, and therefore, 23 HV and 15 OA participants were considered for reliability analysis. All 29 HV and 15 OA participants’ data were used for validity testing. A detailed description of the participant demographics, anthropometric data, physical activity levels, and testing data pain intensity are presented in Table 1.

**Table 1:** Demographics and clinical characteristics of all participants and separated by group.

Participants Characteristics	Total (N = 44) *(Mean ± SD/N)*	HV (N = 29) *(Mean ± SD/N)*	Knee OA (N = 15) *(Mean ± SD/N)*
Age (years)	31.6 ± 11.2	28.4±7.4	37.6 ± 13.4
Height (cm)	168.2 ± 8.6	168.3 ± 6.9	168 ± 11.4
Weight (kg)	73.8 ± 20.6	68.3 ± 11	84.4 ± 29.8
Gender (F, M)	28 F, 16M	19 F, 10 M	9 F, 6 M
Dominant leg (Right)	39	26	13
NPRS (0–10 cm)	0.4 ± 1.2	0 ± 0	1.1 ± 1.8
SGPALS	2.9 ± 0.8	3.1 ± 0.7	2.5 ± 1
Days between sessions	10 ± 3	12 ± 3	9 ± 4

OA = Osteoarthritis, NPRS = Numerical pain rating scale, SGPALS = Saltin-Grimby Physical Activity Level Scale

### Reliability

#### Test-retest reliability of novel device

The test-retest reliability of the ND (Table 2) ICC_(2,3)_ was 0.97 (with narrow CIs) for all participants and did not change after group separation. The SEM was 53 N, and MDD_95_ was 148 N when all participants were included.

**Table 2:** Test-retest reliability of novel device (within-day, intra-rater) of all participants and separated by group.

Subjects	Fmax (N) *Test 1*	Fmax (N) *Test 2*	SD_pooled_ (N)	ICC *(95% CI)*	SEM N (%)	MDD_95_ N (%)
**All**	414.2	425.1	307.1	.969 (.940, .983)	53 (8.1)	148 (22.3)
**HV**	464.5	474.3	194.2	.983 (.959, .993)	34 (5.0)	93 (13.8)
**OA**	350.3	349.7	142.2	.947 (.848, .982)	29 (10.4)	80 (29)

All = All participants, HV = Healthy volunteers, OA = Osteoarthritis, ND = Novel Device, Fmax = Mean maximum force, N = Newtons, % = Percent of Mean Force, SD_pooled_ = Average standard deviation, ICC = Interclass Correlation Coefficient, CI = Confidence interval, SEM = Standard error of mean, MDD_95_ = Minimal detectable difference

#### Within- and between-day interrater reliability

Table 3 provides the within-day and between-day *interrater* reliability (ICC, CI), including the group mean Fmax values, SEM, and MDD_95_ of all participants and HV and OA groups separately.

**Table 3:** Within-day and between-days interrater reliability of the hand-held and novel devices for all participants and separated by group.

**Within-day Interrater Reliability**							
**Subject**	**Device**	**Fmax (N) Rater 1**	**Fmax (N)** **Rater 2**	**SD**_**pooled**_ **(N)**	**ICC**_**2,3**_ **(95% CI)**	**SEM N(%)**	**MDD**_**95**_ **N(%)**
**All**	**HHD**	406.6	395.4	95.9	.881 (.631, .882)	33 (8.1)	92 (22.5)
	**ND**	430.4	411.1	177.2	.928 (.864, .962)	48 (11)	132 (30.6)
**HV**	**HHD**	443.1	428.8	75.7	.798 (.531, .914)	34 (7.6)	94 (21.2)
	**ND**	481.1	464.8	162.1	.905 (.779, .960)	50 (10.3)	139 (28.7)
**OA**	**HHD**	372.5	361.8	79.6	.892 (.690, .962)	26 (7)	73 (19.4)
	**ND**	380.9	356.3	155.5	.935 (.819, .977)	40 (10.3)	110 (28.8)
**Between-day Interrater Reliability**							
**All**	**HHD**	409.2	416.1	86.7	.869 (.775, .924)	32 (7.5)	87 (20.9)
	**ND**	440.9	438.8	178.6	.885 (.802, .933)	61 (13.8)	168 (38.2)
**HV**	**HHD**	427.6	439.5	84.1	.870 (.749, .933)	31 (6.8)	85 (19.1)
	**ND**	469.4	441.9	178	.928 (.859, .963)	48 (10.8)	133 (30.1)
**OA**	**HHD**	369.1	365.0	75.3	.788 (.402, .924)	35 (9.4)	97 (26.3)
	**ND**	378.9	431.9	172.6	.780 (.416, .919)	81 (18.7)	224 (51.9)

All = All participants, HV = Healthy volunteers, OA = Osteoarthritis, HHD = Handheld dynamometer, ND = Novel device, Fmax = Mean maximum force, N = Newtons, % = Percent of mean force, SD_pooled_ = Average standard deviation, ICC = Interclass Correlation Coefficient, CI = Confidence interval, SEM = Standard error of mean, MDD_95_ = Minimal detectable difference

#### Within-day interrater reliability

The within-day *interrater* reliability of all participants was ICC_2,3_ = 0.88 for the HHD and ICC_2,3_ = 0.93 for the ND. In all analyses, the HHD had a wider ICC CI than the ND. Separate group analysis reveals that the reliability of the HHD was higher in the OA group compared to the HV group. In contrast, reliability of ND was not different between OA and HV groups. The results also showed that the HHD has a higher ICC value in the OA compared to the HV group. When all participants were considered, the measurement from ND had a greater SEM and MDD_95_ compared to HHD, principally due to the larger pooled between subject SD when using the ND. Similarly, in both the groups, the SEM and MDD_95_ values were lower for HHD than ND.

#### Between-day interrater reliability

The *interrater* reliability values were good for both the HHD (ICC_2,3_ = 0.87) and ND (ICC_2,3_ = 0.89), across all participants. However, in the separate group analysis, both devices demonstrated lower reliability in the OA group, than the HV. In all analyses, the HHD had a wider ICC CI than the ND. Similar to the within-day *interrater* reliability, all participants had lower SEM and MDD_95_ values with the HHD than the ND.

### Validity

**Fig 6:**
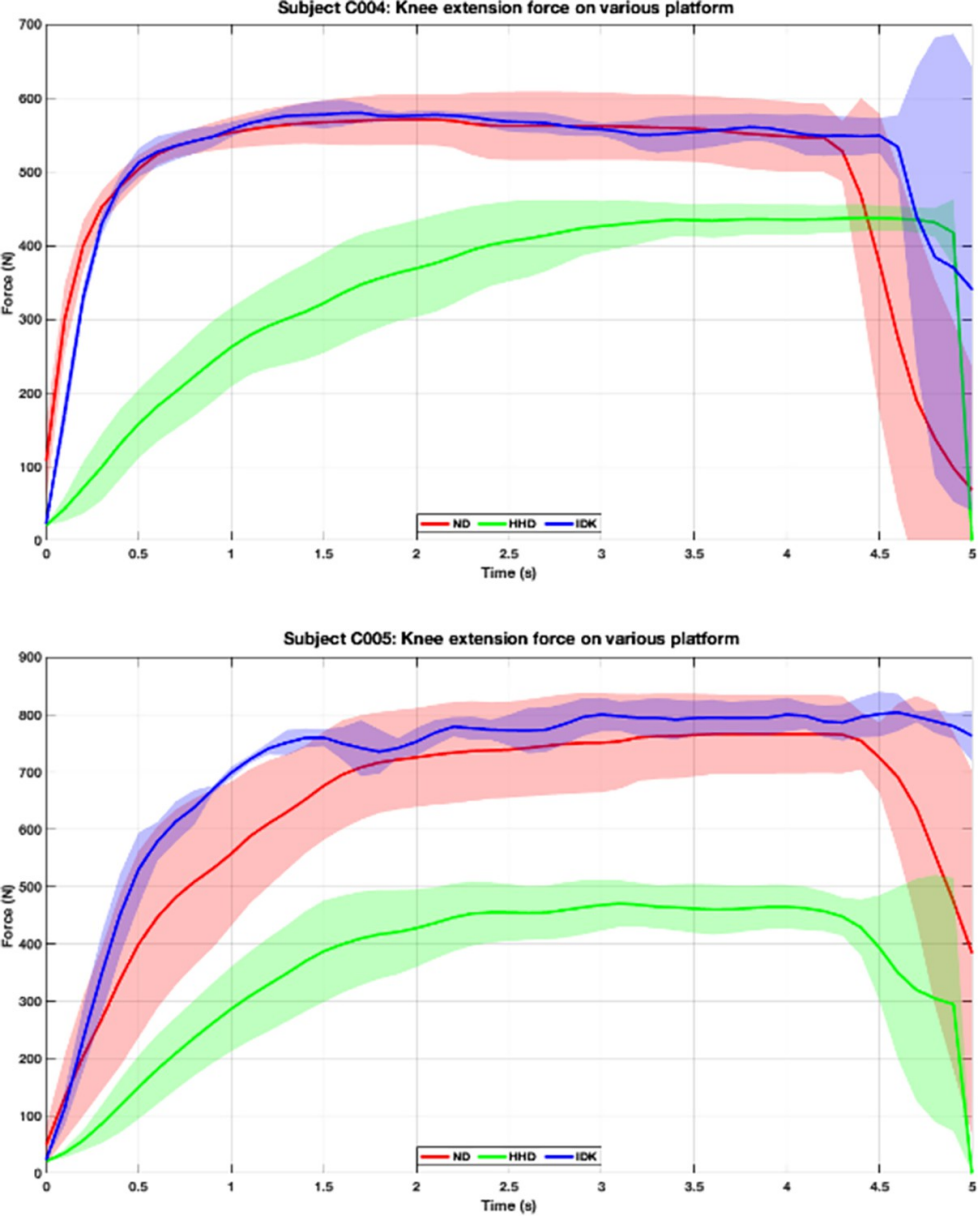
Example force-time curves. Representative time-force curves, averaged across three trials, for two participants (time on the X-axis and force on the Y-axis). The force-time curve mean and standard deviation (shadow) of the ND (red) are similar to those of the IKD (purple). However, the HHD force-time curve (green) differs from those of ND and IKD.

One-way repeated measure ANOVA (Table 4) indicates a statistically significant difference in maximum force generated across the three devices (p < 0.001). Pairwise probability comparison demonstrates differences between the HHD and IKD (p < 0.001, MD_abs_ = 58 N), while no statistically significant difference was found between the HHD vs. ND and ND vs. IKD. Pearson correlations reveal the highest correlation between ND vs. IKD (r = 0.81) compared to the correlation between HHD vs. ND (r = 0.72) and HHD vs. IKD (r = 0.74). Force-time curves (representing force generation patterns across time) for each participant (Fig 6) were correlated between each pair of devices and then averaged. We found the highest correlation average between the ND vs. IKD (0.96 ± 0.05).

**Table 4:** Statistical procedures and outcomes for concurrent validity testing all participants.

Statistical Procedure	HHD vs ND	HHD vs IKD	ND vs IKD
One-way repeated measure ANOVA Pairwise comparison (p-value)	0.267	<0.001	0.051
**Pairwise mean difference (HHD-ND; HHD -IKD; ND-IKD) of Fmax (Newtons)**	-24	-58	-34
**Pearson correlation (r)**	0.717	0.742	0.809
**(95% CI)**	(0.534, 0.836)	(0.571, 0.851)	(0.674, 0.892)
**Force-Time curves Correlation (r) (Mean±SD)**	0.82 ± 0.19	0.83 ± 0.19	0.96 ± 0.05

ANOVA = Analysis of covariance, HHD = Hand-held dynamometer, ND = Novel Device, IKD = Isokinetic dynamometer, p<0.05 for statistical significant threshold, r = Pearson correlation coefficient, CI = Confidence interval

**Fig 7:**
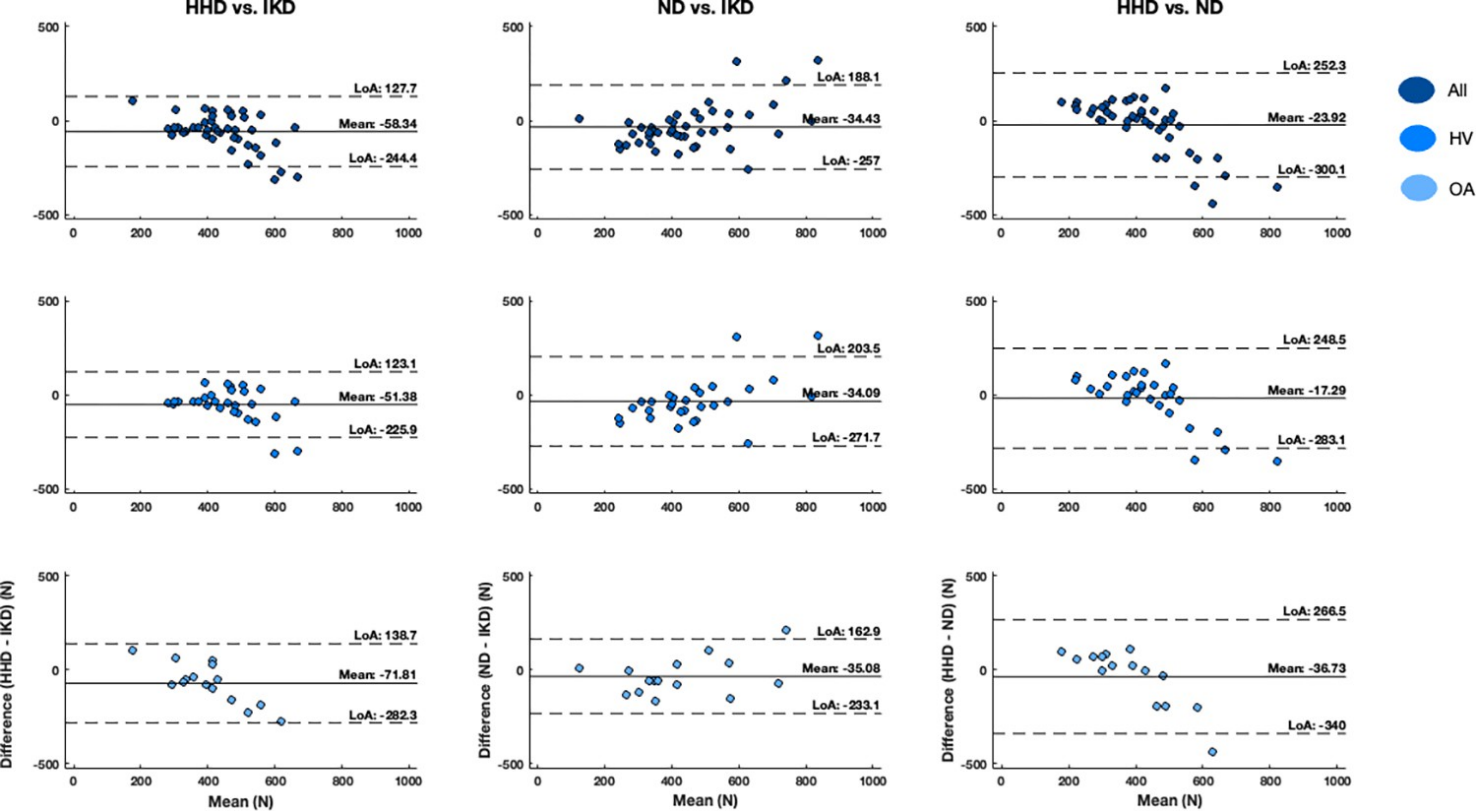
Bland-Altman plots. These represent the mean difference, p-value, limits of agreement (LoA), and bias between each pair of devices. The upper row plots include all participants, the middle row the HVs, and the bottom row knee OA participants. Each graph represents the mean difference at the center (solid line), the upper and lower limits of agreement, (dashed lines), and the dots represent the between device difference in Fmax for each participant.

Bland-Altman plots (Fig 7) indicate systematic bias of the HHD when compared with IKD and ND. The plots also display proportionate bias when comparing HHD with IKD and ND as the HHD progressively increases the underestimation of participant force starting around 450 N. Systematic bias was slight, and no proportionate bias was observed between ND and IKD. The LoA ranges were fairly large between all the devices due to the sample’s between-subject variability.

## Discussion

The overall results denote that the ND has excellent device reliability for measuring knee extensor isometric strength within a session using the same rater. The within-day and between-day *interrater* reliability of the HHD was good, whereas good to excellent reliability was detected for the ND. The findings indicate that the ND demonstrates higher concurrent validity against the gold standard (IKD), while a significant difference in force and proportional bias was observed between HHD and IKD.

### Test-retest device reliability

To objectively measure and document the response to certain rehabilitation interventions such as exercises and manual therapy, muscle strength assessment using a HHD or IKD is common. For those clinics without an IKD, testing larger muscle groups presents a challenge for obtaining accurate and consistent results. Study findings consistently report that the removal of rater-generated resistance from the strength testing protocol improves within-day and between-day reliability [29, 45–47]. Our ND eliminates the need for tester resistance by utilizing equipment already found in most clinics. We inserted a tension load cell into a weight stack machine and took advantage of the machine’s adjustability, ease of patient stabilization, and ability to select joint positioning thus creating a hands-free strength testing unit. Our ND demonstrated excellent *intrarater* test-retest reliability (ICC_2,3_ > .90) across both HV and participants with OA.

### Within-day reliability in healthy individuals

Most studies using HHD for knee strength testing report only within-day *intrarater* reliability. Studies of conventional HHD protocols (manual resistance) reported good to excellent within-session *intrarater* test-retest reliability (ICC = 0.70–0.92) for knee strength in healthy young adults [21, 27]. Researchers have approached the problem by using a belt to anchor the HHD or mounting it to a frame attached to a bench or chair and demonstrated that using their frame-mounted system resulted in good to excellent (ICC = 0.89–0.98) within-day *interrater* reliability [34, 45–47]. In a within-day test-retest study that mounted the HHD to PVC, they reported excellent *intrarater* (ICC = 0.93) reliability in 15 young, healthy individuals [39]. A study by Sung et al. modified their HHD protocol by anchoring the HHD to a frame but had the tester manually stabilize the pelvis during testing of knee extensor strength and reported excellent *intrarater* test-retest reliability (ICC = 0.98) [41]. The ND in our study demonstrated excellent (ICC_2,3_ > .90) within-day *intrarater* test-retest reliability in both HV and OA participants, and the narrow CIs suggest the ability of the device to capture reliable data even in participants with greater strength.

In clinics, multiple examiners often gather data for intervention progression decisions and documentation. Variability among clinical testers has been commonly reported in the literature. The potential for inconsistency among testers necessitates a device that minimizes sources of error to make it suitable for use in research and practice [67, 68]. Therefore, a majority of this study specifically focuses on *interrater* reliability to illustrate a worst-case scenario within the clinic. We found that the within-day *interrater* reliability of the HHD was good when the HV and OA participants’ data were analyzed together. However, the reliability of the HHD was lower in HV participants compared to knee OA, implying that the amount of force generated by the participants plays a crucial role in HHD reliability. Further, a wider CI was observed in the HV compared to OA individuals, again suggesting the testers’ strength could influence measurement *interrater* reliability. We found a study by Sung et al., that reported within-day *interrater* reliability with a HHD as excellent (ICC = 0.98) which they achieved by mounting the HHD to an external frame [41]. This suggests it is necessary to move beyond manual resistance to a system that augments participant and joint positioning and dynamometer stabilization.

### Between-day reliability in healthy individuals

In clinical settings, knee extensor strength assessment is an essential indicator of response to rehabilitation and requires testing during different treatment sessions. Variability in quantitative measurements increases, and the agreement reduces when testing on separate days. Therefore, addressing the between-day *interrater* reliability contributes to the uniqueness and acceptability of devices. Only a few studies reported between-day reliability of conventional HHD testing and only in healthy individuals with studies reporting good to excellent *interrater* (ICC = 0.77–0.98) reliability between sessions [27, 32, 33, 69]. In parallel, the present study found good reliability using the HHD with manual resistance in HV, when protocol repeatability, participant stabilization, and tester positioning were optimized. On the other hand, the ND demonstrated excellent between-day *interrater* reliability in HV with a tight CI and good reliability in participants with OA. Comparing our SEM and MDD_95_ values to prior studies reporting between-day reliability is complicated by the tester differences and participant samples used. We found one paper reporting between-day *interrater* reliability for knee extensors using a dynamometer and strap and a few between-day *intrarater* studies reporting these values and only in healthy participants. Morin et al. reported a between-day interrater reliability of (ICC = 0.95) with an SEM 1.69% (2.47 Nm) and MDC_95_ 4.69% (6.84 Nm), which represent a lower percent of the mean than our reported values [33]. Mentiplay et al., reported between days *intrarater* values ranging between 7.7 to 8.7% of the mean force for their SEM and 21.4 to 24.2% of the mean for the MDC_95_ using a HHD with manual resistance for testing knee extensor strength [32]. These values compare to the SEM of 6.8% and MDD_95_ of 19.1% in our study for *interrater* HHD. In studies where the HHD was fixed by a belt, a lower percentage of mean values were reported for the SEM 5.21% and MDC_95_ 14.78% [38]. Martins et al. also performed a between-day *intrarater* study reporting an SEM of 12% and MDD_95_ of 34% for a HHD mounted to the arm of an IKD system [36]. This situation more closely matches our ND in which we found an SEM of 10.8% and MDD_95_ of 30.1%. The higher MDD_95_ in our study is partially driven by our HV large between-subject variance, which we attribute to the concurrent validity demonstrated by the ND. For comparison, we found one study that reported percent of mean force SEM and MDD_95_ values of 5.7% and 15.7%, respectively, when measuring knee extensor strength with an IKD [32].

### Within and between-day reliability in a clinical population

Few studies assessing knee strength in a clinical population have been conducted that reported *intra-* and *interrater* reliability when using a HHD. Studies testing *intrarater* knee extensor strength within-day were located using participants with OA (ICC = 0.92–0.97) and hematological malignancy (ICC = 0.94) [40, 70]. Another study reported excellent knee extensor muscle strength reliability using a HHD within-day *intrarater* (ICC = 0.98) and *interrater* reliability (ICC = 0.95–0.96) in an unspecified rehabilitation sample [71]. Additional studies conducted in arthritic populations (e.g., TKR, THR, OA) modified their HHD protocols and reported that their redesigned system enhanced (ICC = 0.92–0.99) *interrater* reliability during a single session [35, 40]. The potential drawback of developing individual methods for improving HHD reliability of strength measurements in larger muscle groups, is the potential lack of consistency across raters and the time needed to set up the testing protocol within the clinic setting. The present study found good within or between-day *interrater* reliability using the HHD in a tightly controlled protocol, while the ND demonstrated excellent within-day or good between-day *interrater* reliability in participants with OA, with easier setup.

### Concurrent validity

Most prior research identified that HHDs underestimate the force compared to the gold standard (IKD). A study in older adults found approximately 21 Nm and 27 Nm reduced torque by conventional HHD compared to IKD on the left and right knee extensors, respectively [69]. About a 15 Nm difference in knee extensor torque was found even after altering the participant’s position to supine lying with the knee in 35⁰ of flexion, which provided better leverage to the tester, offering resistance [42]. A few studies used belt-fixed HHD to minimize the role of the tester’s resistance and discerned that the HHD either underestimated or over-estimated the force compared to IKD [37, 43, 44]. Bohannon et al. reported a 35 Nm underestimation in knee extensor torque in HV [43]. Similarly, another study detected 230 N less knee extensor force obtained with the HHD in healthy individuals [44]. In contrast, Lesnak et al. showed belt stabilized HHD overestimates torque 19.4 Nm on average, and the difference was above the reported MDD [37]. However, the lack of adequate proximal segment stabilization owing to the possibility of synergistic muscle groups contributing to the recorded force production could account for overestimations. In addition, a higher possibility of dynamic movement is suspected during some modified and manually resisted HHD strength protocols, risking measurement consistency and validity. We observed significantly lower forces generated during HHD (58 N) testing compared with IKD, while no statistically significant difference in force was found between the ND and IKD (34 N). The differences in force generated by HHD and ND compared to IKD were below MDD_95_, and the ND more closely matched that of the gold standard. One of the primary reasons behind the higher SEM and MDD_95_ for the ND is the greater variability in participant generated force, particularly in the HV, as the ND does not appear to demonstrate a ceiling effect or proportional bias as noted in the HHD vs IKD Bland-Altman plots. The HHD increasingly underestimated the force generated by the participants starting around 450 N. This is a major limitation of the use of a HHD with manual resistance in clinical settings, especially when measuring the strength of large muscle groups and in participants with greater strength [43]. In contrast, the ND did not display proportionate bias, suggesting its validity for application in large muscle group testing and its capture of true isometric strength.

We found a strong correlation among maximum force values obtained by all the instruments; however, the highest correlation coefficient was demonstrated between the ND and IKD. Earlier studies of HV focusing on younger and older participants documented a strong correlation (0.78–0.92) with IKD either by using the conventional HHD technique or when placing the HHD on the tibial pad of a weight stack [32, 36, 42]. The possible reason behind a greater correlation between HHD and IKD in those studies, in contrast to our research, might be attributed to methodological differences such as manual resistance, unstandardized stabilization of the proximal segment, and or positioning of the participants. Another factor likely contributing to a lower correlation of the maximum force produced in our study is between participant variability in our data, as both HV and the clinical population were included in the validity analysis. A potential reason for the lower force generation and less agreement when using the HHD could be pain or discomfort reported at the tibial contact site with the HHD [34]. In our study, 34/44 individuals reported contact site pain during HHD testing, whereas only 19/44 and 11/44 participants reported tibia contact discomfort during testing with the ND and IKD, respectively.

Diverging from other currently available literature, we also assessed the force-time series correlation. We found the highest correlation between ND and IKD with a small SD which illustrates that the force generation pattern matched well with the IKD. This finding suggests that in those clinics without an IKD, the ND could be an efficient means for evaluating rate-of-force/torque development, which is a variable of interest in strength recovery metrics [72].

In fitness settings and clinics without IKD, but with weight stack machines, use of the ND can expand strength testing capabilities of larger muscle groups without significant increases in practitioner time or client burden. The typical testing time using the ND, inclusive of set up, 2–3 warm-up repetitions, and 3 test trials on each limb, is generally 15–20 minutes and allows for more consistent testing positions and easier stabilization of clients than using a HHD. The reduced ND setup time and less complicated software shorten the testing time by15-20 minutes in comparison to an equivalent IKD protocol while providing comparable reliability and validity. Practitioners should be sure to consistently test using the same weight stack machine when assessing patients over time, as different weight stack machines could produce slightly different results due to manufacture designs and specifications.

There were several limitations to this study. First, the major limitation of the ND compared to the IKD is that its application is limited to isometric testing. Second, our study is limited to the reliability and validity of knee extensor isometric strength. At the same time, we believe our findings will translate to testing other large muscles (e.g., hip extensors, hamstrings, hip abductors). However, our device was not tested on other muscle group specific weight stack machines. Third, given our participant directions to gradually build force during the test, we could not demonstrate our device’s ability to provide accurate rate force development data. However, given the high correlation (and tight SD) between the ND force-time curves with that of the IKD, we see no reason why force-time curves from the ND would demonstrate a vastly different rate of force development value from those of the IKD. Fourth, our testers were all fit males. Thus, the findings of the HHD reliability and concurrent validity may not translate to all clinicians or fitness professionals using the HHD as described in our study.

## Conclusion

The study supports both within- and between-day *interrater* test-retest reliability, and maximum force and force-time curve validity of this ND and testing paradigm. The findings suggest that when testing knee extensor force production, the ND is comparable to an IKD and may be superior to HHD in healthy individuals and those diagnosed with mild to moderate knee OA. The HHD protocol used in this study resulted in a ceiling effect, unlike the ND and IKD, indicating that using an HHD to measure larger muscle groups may not fully represent a participant’s strength and is dependent on the resistance offered by the tester. Excellent device test-retest reliability and validity, portability, safety, and the hands-free protocol made possible by the ND suggest potential utility within physical therapy and fitness settings for strength testing large muscle groups such as knee extensors.

## List of abbreviations

CI: Confidence Interval
Fmax: Mean maximum force
GUI: Graphical user interface
HHD: Hand Held Dynamometer
HV: Healthy volunteers
ICC: Interclass Correlation Coefficient
IKD: Isokinetic Dynamometer
LoA: Limits of Agreement
MDD: Minimal detectable difference
MDabs: Mean Absolute Difference
N: Newtons
ND: Novel Device
NPRS: Numerical Pain Rating Scale
OA: Osteoarthritis
SD_pooled_: average standard deviation
SEM: Standard Error of Mean
THR: Total Hip Arthroplasty
TKR: Total Knee Arthroplasty;
US: United States
